# Advances in Ultrasonic Rehabilitation

**DOI:** 10.3390/s26082428

**Published:** 2026-04-15

**Authors:** Vytautas Ostasevicius, Vytautas Jurenas, Laura Kizauskiene, Agne Paulauskaite-Taraseviciene, Joris Vezys, Algimantas Bubulis, Arnas Nakrosis

**Affiliations:** 1 Institute of Mechatronics, Kaunas University of Technology, 51424 Kaunas, Lithuania; vytautas.jurenas@ktu.lt (V.J.); algimantas.bubulis@ktu.lt (A.B.); 2 Department of Computer Sciences, Faculty of Informatics, Kaunas University of Technology, 51368 Kaunas, Lithuania; laura.kizauskiene@ktu.lt; 3 Artificial Intelligence Excellence Centre, Kaunas University of Technology, 51423 Kaunas, Lithuania; agne.paulauskaite-taraseviciene@ktu.lt; 4 Department of Mechanical Engineering, Mechanical Engineering and Design Faculty, Kaunas University of Technology, 51424 Kaunas, Lithuania; joris.vezys@ktu.lt; 5 Department of Applied Informatics, Faculty of Informatics, Kaunas University of Technology, 51368 Kaunas, Lithuania; arnas.nakrosis@ktu.lt

**Keywords:** red blood cells (erythrocytes), aggregation, dissociation, low-frequency ultrasound, acoustic shear forces, erythrocyte biomechanics

## Abstract

The fundamental differences between high- and low-frequency ultrasound for medical purposes were demonstrated. A model describing the effect of ultrasound on erythrocyte aggregation was developed, and the rapid movement of erythrocytes toward the nodes of a standing acoustic wave was demonstrated, with its velocity compared to the rate of erythrocyte dissociation under the influence of shear forces. The *t*-test was used to assess the statistical significance of differences between two blood samples and confirmed the effect of low-frequency ultrasound intensity on erythrocyte aggregation. The study employed a patented low-frequency ultrasound transducer generating a traveling acoustic wave that produces shear forces capable of disrupting erythrocyte aggregates into individual erythrocytes. Since the developed technique is intended for human therapy, it is assumed that the proposed low-frequency ultrasound parameters are safe for flowing blood. Due to deeper and more precise penetration of the acoustic signal into tissues, this ultrasound transducer may be promising for improving microcirculation and alleviating patient condition without medication, as well as for reducing blood pressure and heart rate. The developed technique also enables more effective disruption of heart valve plaques and shows therapeutic potential for tumor treatment and in vivo drug encapsulation. Since erythrocytes in diabetic patients are stiffer than those in healthy individuals, their passage through capillaries is more difficult. Therefore, the developed and patented ultrasound-based sole stimulation technique may produce a positive physiological effect by stimulating blood flow in the capillaries of patients with foot ulcers.

## 1. Introduction

The field of ultrasound rehabilitation is rapidly developing, and therapeutic ultrasound (US) technologies have significantly advanced and are being applied in various medical fields. US wave frequencies greater than 20 kHz have a significant impact on blood hemodynamics. In this study, particular attention is given to the application of US for the dissociation of erythrocyte aggregates.

The separation of microparticles is important in modern biomedical technologies. Currently available techniques for erythrocyte dissociation based on centrifugal sedimentation, magnetic, plasmapheresis, or dialysis require expensive medical equipment and have limitations related to particle quantity requirements. In addition to the widely used centrifugal sedimentation of erythrocytes, the method of magnetic separation in a microchannel has recently gained attention [1]. In this approach, particles with higher iron oxide content, such as erythrocytes, are transported by a ratcheting mechanism using magnetically soft micro-pillars in combination with a directionally cycled rotating magnetic field to dynamically modify the potential energy landscape. Another approach is presented in [2], where laterally driven continuous dielectrophoresis micro-separators are used to separate red and white blood cells suspended in a highly conductive, diluted whole blood. Continuous micro-separators allow blood cells to be dissociated by the lateral dielectrophoretic force generated by a planar array of interdigitated electrodes arranged at an angle to the direction of flow.

The acoustic radiation force-based method described in [3] utilizes differences in particle compressibility to improve the efficiency of erythrocyte dissociation. In this approach, separation efficiency is defined as the fraction of particles collected at the central outlet. The technology presented in this paper allows solving the problem of embolization with the possibility of separating erythrocytes from lipid particles. Since the particles are erythrocytes and lipid droplets in plasma, the erythrocytes gather in the pressure node (in the center of the channel), whereas lipid particles gather in the pressure anti-node (near the side walls).

The erythrocyte sedimentation rate is one of the oldest medical diagnostic tools. However, there is still ongoing debate about the structure formed by the cells during the sedimentation process. In study [4], a direct investigation of the structures formed by erythrocytes in the blood during sedimentation was conducted. It was proven that erythrocyte sedimentation occurs as a dynamic compression of a colloidal gel with plasma channels. Blood cells are suspended in a yellowish substance called plasma. Plasma consists of water and various dissolved molecules. Together, the components of blood plasma account for its large volume in the blood (±55%). Blood separation by plasmapheresis can be used to replace unhealthy plasma in patients with healthy donor plasma [5]. Removal of erythrocytes from whole blood is an essential step in sample preparations intended for biomedical analysis and clinical diagnosis. To address the limitations of current methods, such as centrifugation and chemical lysis, a novel microfluidic device for high-efficiency erythrocyte removal and leukocyte separation from bulk flows of highly concentrated erythrocytes using a viscoelastic non-Newtonian fluid has been proposed in [6].

The review in [7] describes various methods for attaching nanoparticles and drugs to the erythrocyte surface and discusses the key factors that influence the stability and circulation properties of the erythrocyte-based delivery system in vivo. Paper [8] presents protocols for blood collection, separation of leukocytes from whole blood by erythrocyte lysis, isolation of mononuclear cells by density gradient separation, and various non-flow sorting methods, such as magnetic bead separation, for the enrichment of specific cell populations prior to flow cytometric analysis.

Attention should be paid to US techniques that allow deeper bio tissues to be affected. Different types of US transducers are available, depending on factors such as piezoelectric crystal arrangement, footprint, and frequency. A horn-shaped Langevin ultrasonic transducer was investigated in [9] to better understand the role of the acoustic profile in creating a stable trap. The propagation of US waves in an elastic body was further examined in [10]. The study discusses the characteristics of US wave propagation in an isotropic elastic solid material due to the radial mode excitation of a piezoelectric disk actuator attached to its surface. The authors in [11] investigate the biological effects of low-intensity US in vitro and review the factors that may enhance or inhibit these effects. The lowest possible US intensity required to induce cell death or produce free radicals was determined. The aim of the study [12] was to evaluate whether low-frequency US can be used to detect air trapping in chronic obstructive pulmonary disease. In addition, the ability of low-frequency US to detect the effects of short-acting bronchodilators was evaluated.

After reviewing the literature sources, the conclusion is drawn that there is no evidence regarding the excitation of higher oscillation modes of US transducers that could increase acoustic signal penetration or acoustic pressure while enabling more precise targeting of therapeutically affected tissues. Furthermore, there is also a lack of work related to noninvasive activation of blood hemodynamics.

## 2. Methods

### 2.1. High-Frequency Ultrasound Action

Standing acoustic waves generated by high-frequency US blood vessels present promising opportunities for advancing medical diagnostics and treatments. Since the diameter of an erythrocyte (RBC) does not exceed the acoustic wavelength (Rayleigh’s mode), the acoustic wave radiation force condenses single erythrocytes into aggregates [13].

The acoustic pressure on erythrocytes was modeled using the COMSOL Multiphysics 6.1 software by evaluating:

- The power of acoustic radiation, thanks to which the movement of erythrocytes is directed to a region of stable acoustic pressure.
- The Stokes force, which is caused by friction between the erythrocyte surface and the surrounding plasma, regulates the rate of erythrocyte transport [14].
- The Bjerknes strength, which arises from acoustic radiation emitted by neighboring erythrocytes, and which alters the trajectories of erythrocytes and promotes their aggregation at standing acoustic wave nodes [15].
- The influence of gravity in the acoustic pressure node, which contributes to the settling of erythrocytes when the motion is interrupted, or causes deviations from a straight path when erythrocytes migrate towards the nearest pressure minimum.
- The Bernoulli strength, which arises from the reduced pressure in the extracellular space, further affects the interaction of erythrocytes in the flow field [16].

A standing acoustic wave exerts an acoustic radiation force on erythrocytes within the medium. This force originates from the interaction between the acoustic pressure field and the erythrocyte structures. At the onset of exposure, elastic erythrocytes are uniformly distributed in a steady acoustic field. As acoustic radiation and hydrodynamic drag forces arise, the erythrocytes begin to migrate from their initial positions.

The nonlinear radiation force *F*_rad_ acting on erythrocytes can be defined as the time-averaged second-order force evaluated on a fixed surface ∂*Ω* surrounding the erythrocyte in an inviscid fluid. Under inviscid conditions, the vector *F*_rad_ is expressed as the sum of the time-averaged second-order field of acoustic pressure ⟨*p*_2_⟩ and the momentum flux tensor *ρ*_0_⟨*ν*_1_*ν*_1_⟩ [17].

(1) $$\begin{array}{ll} F_{rad} & =-\int_{\partial \Omega }\mathrm{dr}\left\{\langle p_{2}\rangle n+\rho_{0}\langle \left(n\cdot \nu_{1}\right)\nu_{1}\rangle \right\}\\ & =-\int_{\partial \Omega }\mathrm{dr}\left\{\left[\frac{\kappa_{0}}{2}\langle p_{1}^{2}\rangle -\frac{\rho_{0}}{2}\langle v_{1}^{2}\rangle \right]n+\rho_{0}\langle \left(n\cdot \nu_{1}\right)\nu_{1}\rangle \right\} \end{array}$$

where: *ρ*_0_—plasma density, *v*_1_—acoustic wave velocity, *p*_1_—linear pressure, κ_0_—compressibility coefficient, *n*—normal vector.

The standing acoustic wave radiation force acting on an erythrocyte is a gradient force of the potential function *U*_rad_ [18]:

(2) $$F_{rad}=-\nabla U_{rad}$$

(3) $$U_{rad}=V_{p}\left[Re[f_{1}]\frac{1}{2\rho_{0}c^{2}}\left\langle p^{2}\right\rangle -Re[f_{2}]\frac{3}{4}\rho_{0}\left\langle {\nu_{1}}^{2}\right\rangle \right]$$

(4) $$f_{1}=1-\frac{K}{K_{p}},\;\;\;\;f_{2}=\frac{2\left(\rho_{p}-\rho_{0}\right)}{2\rho_{p}+\rho_{0}}$$

where *ρ_p_*—erythrocyte density, c—speed of sound, *f*_1_ is the dimensionless scattering ratio depending on the compressibility of the erythrocyte and plasma, *Re*—real part.

The scattering rate *f*_2_, which characterizes the translational response of an erythrocyte to the acoustic field, *K* and *K_p_*—are bulk modules of plasma and erythrocytes, respectively, *V_p_*—erythrocyte volume.

A digital model of a piezoelectric actuator cylinder with an internal radius of R = 60 mm was created. The direct piezoelectric effect was electrically excited by the harmonic law at a frequency of *f*_0_ = 350 kHz. The cylinder was filled with a blood suspension that was exposed to standing acoustic waves. A 2D model of the system was analyzed. The following physical parameters are used in the model: *c* = 1.48∙10^3^ m/s is the sound velocity in blood; *ρ_p_* = 4∙10^3^ kg/m^3^ is the particle density; *Kp* = 2.2 GPa is the bulk modulus of the erythrocyte, and *a*_0_ = 7.5∙10^6^ m/s^2^ is the amplitude of the normal acceleration of the transducer.

The equations of direct and inverse piezo effect in the piezoelectric transducer take the form:

(5) $$\begin{array}{c} D=E\varepsilon +eS\\ \sigma =-eE+c_{0}S \end{array}$$

where *D* denotes electric induction in the piezoelectric transducer; *E*—the tension of the electric field; *ε* dielectric constant; *e* piezoelectric coefficient; *S* transverse deformation; *σ* elastic stress; c_0_ the velocity of acoustic deformation waves.

If the piezo transducer operates at resonant frequency, Equation (5) is transformed to

(6) $$\begin{array}{c} D\exp\left(-j\omega t\right)=E\varepsilon +e\frac{\partial U_{0}}{\partial x}\\ \sigma =-eE+\frac{\lambda_{0}\omega }{2\pi }\cdot \frac{\partial U_{0}}{\partial x} \end{array}$$

where *j* denotes imaginary unit; *ω* the resonant frequency; *U*_0_—the displacement; *t*—time; *λ*_0_ = 2πc_0_/ω.

Then the elastic stress in the piezoelectric transducer can be expressed as follows:

(7) $$\sigma =\frac{\lambda \omega }{2\pi }\cdot \frac{\partial U_{0}}{\partial x}-\frac{e}{\varepsilon }\left(D\exp\left(-j\omega t\right)-e\frac{\partial U_{0}}{\partial x}\right)$$

The oscillatory motion of the piezoelectric transducer is described by the following system of equations:

(8) $$\begin{array}{c} \frac{\partial^{2}U_{0}}{\partial t^{2}}=c_{0}^{2}\frac{\partial^{2}U_{0}}{\partial x^{2}}\\ \frac{\partial^{2}U_{1}}{\partial t^{2}}=c_{1}^{2}\frac{\partial^{2}U_{1}}{\partial x^{2}} \end{array}$$

with the following boundary conditions:

(9) $$\begin{array}{c} U_{0}=U_{1}\\ \frac{\lambda_{1}\omega }{2\pi }\cdot \frac{\partial U_{1}}{\partial x}=\frac{\lambda_{0}\omega }{2\pi }\cdot \frac{\partial U_{0}}{\partial x}-\frac{e}{\varepsilon }D\exp\left(j\omega t\right)+\frac{e^{2}}{\varepsilon }\cdot \frac{\partial U_{0}}{\partial x} \end{array}$$

where *U*_1_ denotes the displacement; *c*_1_ the velocity of acoustic deformation waves in the piezo actuator; *λ*_1_ = 2πc_1_/ω.

The COMSOL Multiphysics system was used for the mathematical model using the finite element numerical method. The acoustic fluid pressure was applied to the frequency domain. The boundary of this domain was excited by the normal acceleration of the piezoelectric actuator cylinder elements.

**Figure 1:**
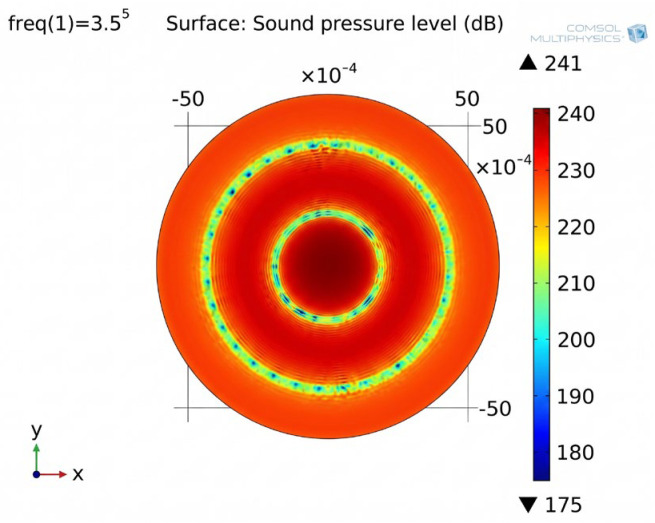
Acoustic pressure level (dB) in blood suspension.

For effective separation of erythrocytes, the acoustic excitation frequency was selected to concentrate erythrocytes into two rings and was set at 350 kHz. The pressure fields of the standing waves in blood suspension are shown in Figure 1.

Figure 1 illustrates that there are three high-pressure regions and two low-pressure regions in the acoustic pressure field. Subsequently, the equation of erythrocyte motion in the acoustic field was solved, allowing the determination of erythrocyte positions within the field at a given time moment *t*_1_. At the initial time *t*_0_, the erythrocytes are uniformly distributed in the acoustic field. When the transducer is excited, the US standing wave induces erythrocyte motion, causing them to migrate and accumulate in those regions of the acoustic pressure field where the sound pressure is minimal.

**Figure 2:**
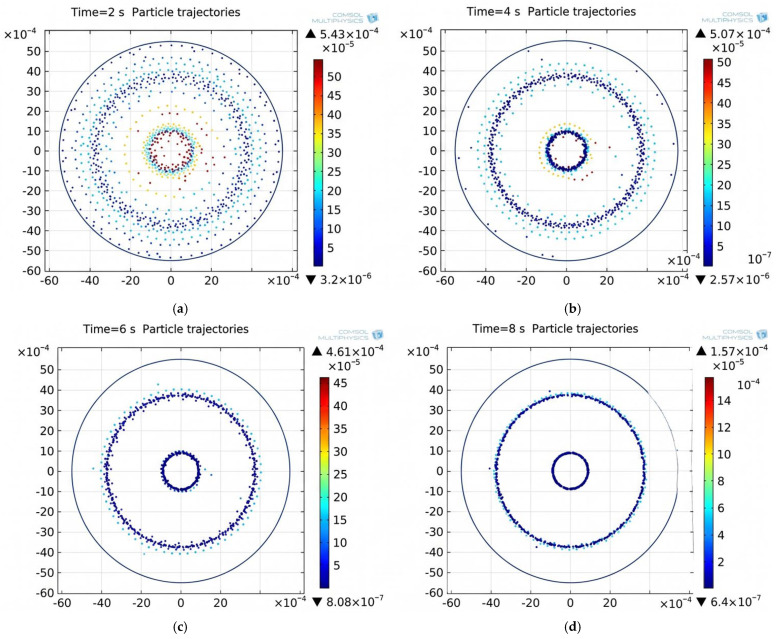
Erythrocytes (particles) distribution in the plasma at different times *t_i_*: (**a**)—2 s; (**b**)—4 s; (**c**)—6 s; (**d**)—8 s.

The evolution of the erythrocyte distribution in the temporal state is illustrated in Figure 2. Colored columns indicate the instantaneous erythrocyte velocity at discrete points *t_i_* obtained by numerical integration of the governing equations of motion.

**Figure 3:**
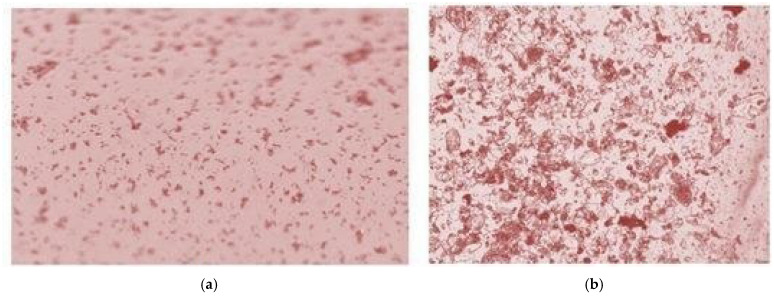
Microscopic view of blood suspension before (**a**) and after (**b**) 350 kHz ultrasound exposure.

Erythrocytes migrated to regions of reduced acoustic pressure within 8 s. This rapid migration enabled stable and reproducible aggregation of erythrocytes in predefined areas of the acoustic field, achieved through controlled adjustment of the vibration parameters generated by the transducer. To evaluate the effectiveness of erythrocyte aggregation in the blood suspension, two separate samples were examined under a microscope. The results are presented in Figure 3. Image (a) depicts a sample of the blood suspension before the aggregation procedure, and (b) illustrates the sample after the aggregation process.

The authors in [19] explain the aggregation of erythrocytes under the influence of high-frequency US. Paper [20] investigated the intensity of erythrocyte aggregation resulting from exposure to high-frequency US in the range of 9–28 MHz. It has been suggested that erythrocyte aggregation may increase blood viscosity due to a reduction in the shear coefficient of the acoustic signal, which may subsequently lead to elevated blood pressure and pulse rate [21]. Increased erythrocyte aggregation has also been associated with inflammatory blood markers in various pathological conditions [22], while a study [23] suggests that erythrocyte aggregation may impair oxygen uptake in blood. On the contrary, low-frequency US is safer because it does not overheat bio tissues by affecting them mechanically through the effect of shear forces created by the traveling acoustic waves. The findings of the study [24] may change our view of the frequent indiscriminate use of diagnostic high-frequency US in clinics. When evaluating the important feature of high-frequency US, focusing on biomedical therapy and diagnosis, one should not forget the undesirable morphological changes in biological tissues because of thermal effects [25].

### 2.2. Low-Frequency Ultrasound Action

Thermal indices widely used in high-frequency diagnostic imaging are not important for low-frequency US therapeutic applications. The effectiveness of US therapy in rehabilitation is not solely determined by the frequency of the US signal. The penetration depth of the US signal is crucial for achieving therapeutic effects. While low-frequency US can penetrate deeper into tissues, the actual penetration depth can vary based on the thickness of the tissue and the specific US frequency used. To prove this, it was first necessary to verify the effect of low-frequency US on blood samples. Human blood samples were obtained using protocols approved by the Kaunas Regional Biomedical Research Ethics Committee. All human subjects signed a consent form approved by the Kaunas Regional Biomedical Research Ethics Committee (No. 2022-03-10 Nr. BE-2-39).

More than 300 blood samples from 42 male patients were sonicated on a blood testing bench at varying ultrasound (US) intensities and exposure times (Table 1). Blood was collected into tube VACUETTE^®^ K2E K2EDTA (Greiner Bio-One GmbH, Kremsmünster, Austria), the inner wall of which is coated with an anticoagulant that prevents the clotting of a blood sample, unlike the blue-top sodium citrate tube, mainly used to collect blood for coagulation tests. All patients were hospitalized, so additional blood was taken before surgery for the experiment. The automated “Swelab Alfa” system was used for blood sample analysis. The blood samples were divided equally into 7 parts and exposed to low-frequency ultrasound. Two blood samples were taken: one as a control sample without ultrasound exposure, and the other sample treated with ultrasound.

Sonication was performed using water bath sonication technology with ultrasonic cleaner CT-400 (Wah Luen Electronic Co., Ltd., Jiangmen, Guangdong, China), at an ultrasound frequency of 46 +/−2 kHz and different ultrasound intensities, and the duration of exposure to ultrasound. To confirm ultrasound frequency, measure ultrasound intensity, and maintain constant parameters during each stage of the experiment, tests were performed with a hydrophone HCT-0320 coupled with an acoustic cavitation meter MCT-2000 (Onda Corp., Sunnyvale, California, USA).

Sonication tests of the blood samples were performed at three different ultrasound intensities of ~120 mW/cm^2^, ~60 mW/cm^2^, and ~10 mW/cm^2^ by changing sonic bath pre-sets on input power.

On the same ultrasound intensity, sonication of the blood was applied in two different sonication times (90 s and 180 s). Since ultrasound with electric power leads to higher temperatures that might degrade the blood samples, the temperature was strictly monitored and controlled in the range of 20–28 °C.

**Figure 4:**
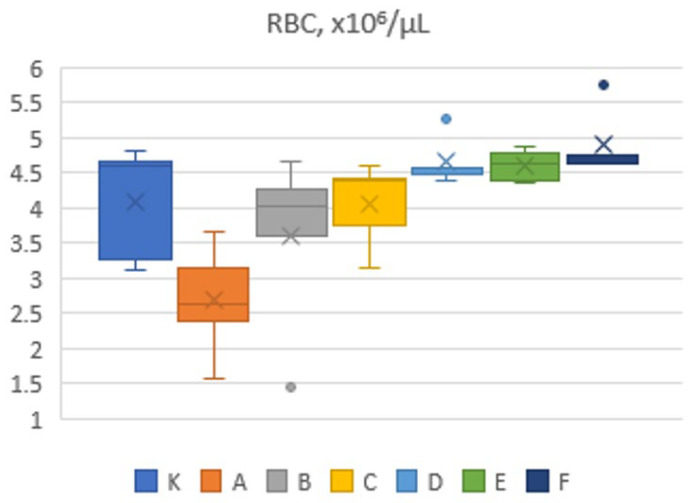
RBC exposed to low-frequency US counts.

The change in RBC (red blood cell count of blood samples exposed to US) is presented in Figure 4, indicating the significant influence of US on this blood parameter.

Each patient’s blood sample was sonicated under six different US intensity-time conditions (A, B, C, D, E, F), while an additional sample of blood was kept unexposed to US (K) and served as a control. To ensure the statistical validity of the analysis, the normality of the data was assessed using the Kolmogorov–Smirnov test. In the cases where significant deviations from normality were identified, appropriate transformations were applied to address this issue. Subsequently, paired *t*-tests were performed to compare group means. When significant differences were observed, an analysis of variance (ANOVA) was conducted to assess the statistical significance of the differences between the two blood samples, providing insights into the impact of the process on the target population. The ANOVA analysis was performed using MATLAB R2018a to analyze the *F*-statistics of the assessed blood parameters. The obtained statistics correspond to an *F* distribution with 40 and 6 degrees of freedom in the numerator and denominator, respectively, F∼F40.6. The results of the multiple comparison test showed that the mean values of the 14 US conditions were significantly different from those of the control group. Higher intensity US demonstrated the greatest effect on the test parameters (Table 2).

Hemolysis is the destruction of erythrocytes and the release of hemoglobin from the cells into the surrounding medium. In our case, it is a non-cellular hemolysis in which the erythrocytes are broken down outside the blood vessel. Normally, the life span of erythrocytes is about 125 days. During hemolysis, the level of bilirubin, a metabolite of hemoglobin, increases in the body. If hemolysis is prolonged, it becomes chronic, characterized by slowly progressive anemia and tissue hypoxia. For these reasons, it was decided to evaluate the effect of US on hemolysis and identify potentially dangerous effects. The test was performed with an Infinite 200Pro device and Tecan i-control V2.0 software at the ultrasonic parameters shown in Table 3.

The results of hemolysis are shown in Table 4.

According to the results of the hemolysis test, only low-intensity US (~10 mW/cm^2^) operated within a safe mode, and no hemolysis was observed after the US exposure. The calculated mean values were: 0.5929 for 6 (SD = 0.2887) and 0.6751 for 7 (SD = 0.1149). Exposures to higher US intensities resulted in hemolysis in the blood samples. However, the test was performed on static blood samples. Under real physiological conditions, where blood is continuously flowing, the results may differ. Therefore, additional experiments are required to evaluate the safety of US exposure in flowing blood.

**Figure 5:**
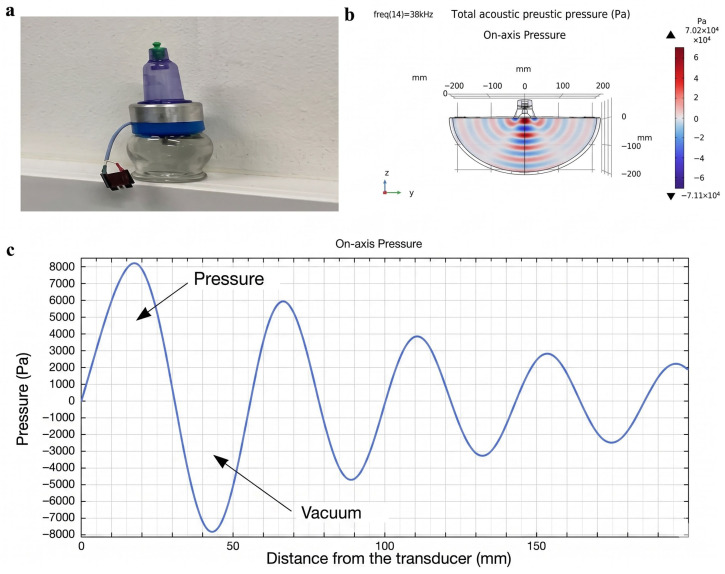
Low-frequency US for rehabilitation: (**a**) low-frequency US transducer; (**b**) acoustic signal deeply penetrating bio tissues; (**c**) shear effect created by a traveling acoustic wave at resonant frequency of 38 kHz.

For the application of in vivo results to human therapy, a low-frequency ultrasonic acoustic wave generation device was developed. The device consists of a medical cup (Acus Med Sp. z o. o, Kolbuszowa Dolna, Poland), a vacuum pump (Acus Med Sp. z o. o, Poland), that enables the cup to adhere tightly to the surface of the human body without the use of sealing gels, ring-shaped piezoelectric elements (Ferropperm A/S PZT-4, Kvistgård, Denmark), and a custom-designed control unit (Figure 5a).

This low-frequency US transducer has been designed for compatibility with human skin and therapeutic applications. It is integrated into a glass cup and operates without the need for US gel and preheating of the cup. This transducer has been patented [26]. The model of this transducer, based on the COMSOL Multiphysics 6.1 program, allowed us to demonstrate how the higher radial oscillation mode of the transducer creates the low-frequency traveling acoustic wave, which penetrates deeper and more precisely into biological tissue (Figure 5b), creating pulsating acoustic pressure (Figure 5c) in it, which detaches (dissociates) erythrocytes from aggregates.

## 3. Discussion

Low-frequency US acoustic waves create acoustic radiation forces and associated fluid shear stresses that can manipulate, deform, and separate erythrocytes from aggregates without necessarily causing their destruction. Human erythrocytes tend to form aggregates, called “rouleaux,” at very low shear rates of up to 10 s^−1^. At higher shear rates, erythrocytes typically dissociate and flow individually within the vessel. Erythrocyte aggregation is influenced by several factors, including hematocrit (the volume fraction of RBCs in the blood), shear rate, vessel diameter, membrane stiffness, and the composition of the suspension medium. The traction forces involved in the formation of the beads are relatively weak. Therefore, the beads can be broken down into smaller fractions or even single cells by applying sufficient shear stress (0.5–30 Pa) generated by controlled traveling acoustic wave devices. The US scattering study of blood was conducted under the assumption that blood essentially behaves as a continuum. Figure 4 shows that, in vitro, exposure to high acoustic pressure (high-intensity US) resulted in a greater formation of erythrocyte aggregates compared to erythrocytes from a control sample that was not exposed to US, as reported in [27]. Since the erythrocytes in the aggregate are counted by the blood analyzer as single erythrocytes, the decrease in RBC count at high-intensity US exposure increases the volume of blood aggregates, reducing their number per unit volume and increasing blood viscosity. However, the change in the number of cells counted can be caused not only by agglutination/disaggregation. For example, exposure to high-intensity US may lead to the destruction of some erythrocytes, which would also result in a reduced cell count reported by the analyzer. The homogeneity of erythrocyte aggregates in the blood depends on the hematocrit and the compression of erythrocytes by the acoustic wave. Our studies show that the changes in erythrocytes and hematocrit following US exposure are identical and depend on the intensity of the US. The attractive forces involved in the creation process of aggregates are relatively weak. Hence, it is possible to dissolve rouleaux into smaller fractions or even into single cells by applying sufficient shear stress (0.5–30 Pa), generated by controlled traveling acoustic wave devices. Excessive shear stresses (>150 Pa) can cause erythrocyte damage, hemolysis, and blood coagulation-clotting. As blood flow in the arteries of living organisms is pulsatile, flow velocity varies periodically according to the cardiac cycle. Blood flow velocity in major arteries differs significantly, with typical values ranging from 30 cm/s in smaller arteries to 500 cm/s at the aortic valve. In the microscopic image showing erythrocyte aggregate formation (Figure 2), rapid erythrocyte movement toward the nodal points of the acoustic field can be observed within 8 s. At a comparable rate, erythrocytes move away from aggregates under the influence of shear stresses induced by low-frequency US. In paper [28], the motion of interacting erythrocytes in shear flow was modeled, demonstrating behavior similar to that observed during erythrocyte aggregation. Bearing in mind that the acoustic signal weakens when penetrating biological tissues, as demonstrated by the attenuation of the acoustic signal transmitted by our device through the sheep’s body from 1000 mW/cm^2^ at the input to 0.24 mW/cm^2^ when the acoustic wave propagates at a distance of 30 cm through the sheep’s biological tissues (skin, bones, muscle-fat tissue and small areas of biological gas) [29]. Considering that the strength of tumor tissue is lower than that of surrounding healthy tissue, it is possible to activate significantly more intense acoustic signals, which, thanks to shear forces, can affect tumors at a greater depth, since the penetration of the acoustic signal emitted by our designed transducer is 4 times greater than that of existing transducers. Analyzing the conditions leading to hemolysis, it can be stated that erythrocytes exposed to US initially separate from aggregates into individual cells within a few seconds. Under prolonged exposure to shear forces, these cells undergo cyclic deformation caused by mechanical stresses and, following membrane rupture, eventually hemolyze. As erythrocytes are the stiffest blood cells, they are susceptible to rupture and hemolysis due to changes in shear stress and osmotic pressure. Therefore, erythrocytes located on the surface of aggregates may be affected by hemolysis, and the intensity of the acoustic signal weakens as it propagates further into the aggregate without hemolyzing it.

The transition from erythrocyte aggregation to disaggregation is influenced by the interaction of plasma and erythrocytes. The bridging mechanism involves the formation of bonds between erythrocytes through adsorbed macromolecules, while the depletion mechanism results from the removal of macromolecules from the extracellular space, resulting in an effective attraction force between cells. Both mechanisms can coexist under experimental conditions, and the relative contribution of each depends on factors such as macromolecule concentration and the synergistic interaction of various physiological parameters. Depletion effects are associated with the finite size of macromolecular aggregation promoters, such as fibrinogen or dextran, and may be affected by changes in ultrasound parameters. These parameters can change the shear conditions of blood plasma, which in turn affects the aggregation and disaggregation behavior of erythrocytes.

Examining the changes in Figure 4, it can be observed that lower US intensities lead to an increase in RBC count. The numerical results presented in [30] show that the acoustic wave acting on the plasma causes recirculation flow, which contributes to the aggregation and deformation of erythrocytes. The dependence of blood pressure on blood viscosity in a group of healthy individuals without risk factors for cardiovascular disease is presented in the article [31]. The results show that systolic blood pressure in healthy individuals is not affected by hematocrit and blood viscosity. On the contrary, diastolic blood pressure is related to hematocrit values. An increase in RBC count with a less intense US signal acts on blood aggregates, dissociating them into smaller or single units of erythrocytes. As a result, the analyzer registers an increase in their quantity, and the gaps created between them lead to a decrease in blood viscosity, which simultaneously reduces blood pressure. In vivo experiments with sheep [29] demonstrated a 13–15% decrease in blood pressure within 7 min of US exposure. Since hemoglobin in single erythrocytes can interact with oxygen across the entire cell surface due to the increased gaps between erythrocytes, oxygen exchange in the blood increases by approximately 10–12% when blood enters the lungs. Consequently, breathing becomes easier, as demonstrated by the in vivo results reported in [29]. In addition, the pulse rate also decreases by approximately 10–12%. The results of this study also show that the acoustic signal penetration of our transducer through biological tissues is 4 times greater than that of transducers currently available on the market. In vivo studies on sheep were conducted as part of a COVID-19-related project for noninvasive therapy of pulmonary hypertension. The results demonstrated improvements in blood physiological parameters. These findings are currently being applied in the development of therapeutic approaches for human pulmonary hypertension. The developed equipment enhances the potential of this technology for critical applications, including heart valve plaque removal, drug encapsulation, tumor therapy, and diabetic foot ulcer rehabilitation. Since the essence of this discovery is related to the activation of hemoglobin molecules, approximately 300 million of which are present in each erythrocyte, it is important to identify whether the hemoglobin content in erythrocytes taken for in vitro experiments corresponds to that in in vivo conditions. According to [32], the results of 28 studies involving approximately 2000 participants suggest that the accuracy and agreement between in vitro and in vivo hemoglobin measurements are acceptable.

### 3.1. Heart Valve Plaque Removal

The human microbiome comprises a wide spectrum of microorganisms that influence human physiology, particularly in relation to the immune system. Upon entering the body, bacteria tend to aggregate into colonies embedded within a biofilm-forming matrix, typically composed of polysaccharides, extracellular DNA, and proteins. This matrix confers significant resistance to antibiotics and other therapeutic agents. Due to its noninvasive and localized mode of action, low-frequency US represents a promising approach for the treatment of bacterial infections. Another clinical challenge is calcific aortic stenosis, which is currently treated by surgically replacing the aortic valve [33,34]. The technique we have developed and patented enables precise and deep targeting of propagating acoustic waves, characterized by the generation of pulsating shear forces. This method may be particularly well-suited for the noninvasive removal of calcified plaques from heart valves.

### 3.2. In Vivo Drug Encapsulation

Erythrocytes are recognized as highly efficient drug carriers [35,36,37]. Currently, the predominant method involves an ex vivo approach, wherein erythrocytes are isolated from the patient’s blood, loaded with therapeutic agents, and subsequently reintroduced into the patient. Erythrocytes are particularly well-suited for clinical applications involving drug encapsulation via hypotonic hemolysis, especially for in vivo drug delivery. Following the dissociation of single erythrocytes by low-frequency US, the conjugation of therapeutic agents to the surface of erythrocytes becomes especially advantageous. Notably, the erythrocyte membrane remains intact during this process, avoiding hemolysis and thereby ensuring prolonged circulation within the bloodstream. Furthermore, this technique facilitates more extensive drug binding across the entire erythrocyte surface, enhancing loading capacity. Drug encapsulation into single erythrocytes is also more straightforward and efficient compared to encapsulation into erythrocyte aggregates, resulting in faster and more effective pharmacological action. The effect of US therapy on hematological dynamics and plasma fibrinogen levels during the inflammatory phase of muscle injury was investigated in a study [38]. US reduced fibrinogen and hemoglobin levels, which reduced the anti-inflammatory effect.

### 3.3. Diabetic Foot Ulcer Rehabilitation

The estimated lifetime incidence of diabetic foot ulceration among individuals with diabetes is approximately 25%. In this population, the probability of lower-limb amputation is significantly elevated and is frequently associated with poor clinical outcomes [39]. To reduce the risk of amputation in patients with diabetic foot ulcers, a device based on acoustic stimulation in the sonic and ultrasonic frequency ranges has been developed and patented [40]. A real image of the actuator is shown in Figure 5.

**Figure 6:**
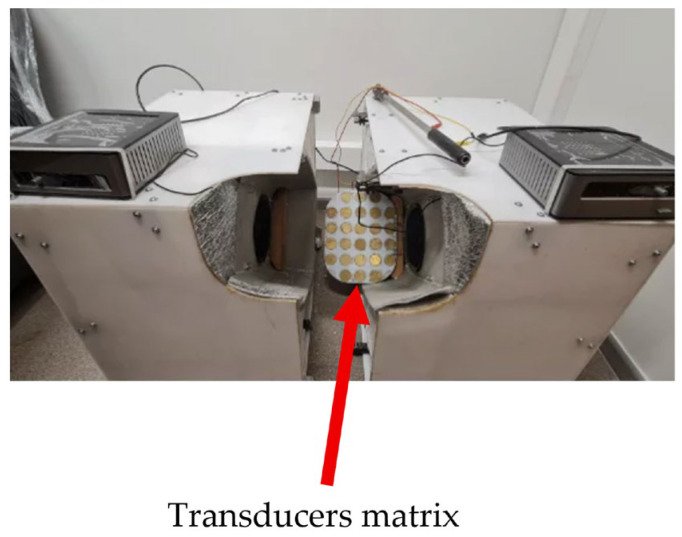
Image of the lower-limb stimulator.

The device shown in Figure 6, similar to that shown in Figure 5, produces analogous hemodynamic changes. The article [41] states that diabetes is associated with many hemorheological changes. Decreased deformability of erythrocytes, increased aggregation, vasoconstriction, increased blood viscosity, and decreased oxygen delivery have a significant impact on the healing of wounds, such as foot ulcers. Basically, there is endothelial dysfunction and permeability changes; this impairs wound healing in diabetic patients. Analysis of microcirculation and hemorheology in diabetes and consideration of treatment methods for diabetic foot ulcers (e.g., hyperbaric oxygen therapy, laser, and vacuum) can help treat patients’ pathologies. All these problems could be solved with the help of our developed and patented devices. Another source [42] states that an alternative to traditional diabetic foot therapy is low-frequency US wound debridement, which accelerates and shortens wound healing due to a larger wound area and a significant reduction in bacterial load.

## 4. Conclusions

The identified effects of low-frequency US on blood parameters demonstrate the potential for rapid, noninvasive therapeutic interventions in both clinical and home settings using the developed technique. Numerical modeling revealed that standing wave US promotes erythrocyte aggregation, whereas low-intensity US—through the propagation of traveling acoustic waves—facilitates the dissociation of erythrocyte aggregates into single cells. This process activates the full surface area of erythrocytes for gas exchange and increases hemoglobin’s affinity for inhaled oxygen. Consequently, the reduced aggregation of erythrocytes contributes to lower blood viscosity, which may lead to decreases in blood pressure and pulse rate. The experimentally validated research results led to the development of a patient-friendly low-frequency US transducer. In this study, the safety limit of US intensity was determined based on the hemolysis threshold. Within the frequency range of 44–48 kHz, hemolysis was not observed at acoustic intensities below 60 mW/cm^2^. The results obtained from the US application support the validity of the proposed assumptions. Furthermore, the developed equipment, which allows the US signal to penetrate deeper into biological tissues without causing thermal effects but instead exerting mechanical action, shows promise across several medical fields, including the removal of plaque from heart valves, in vivo drug encapsulation, tumor therapy, and the rehabilitation of diabetic foot ulcers.

## Figures and Tables

**Table 1:** Low-frequency US acting on blood characteristics.

	Mode	Exposure Time, s	Intensity, mW/cm^2^	Power W	Frequency kHz
K	Control	0	0	0	0
A	High-intensity US	90	100–150	60	48
B	High-intensity US	180	100–150	60	48
C	Medium-intensity US	90	50–70	35	44
D	Medium-intensity US	180	50–70	35	44
E	Low-intensity US	90	5–12	10	44
F	Low-intensity US	180	5–12	10	44

**Table 2:** Repeated measures ANOVA results.

Parameter:	Ultrasound Condition	*p*	Lower	Upper
RBC	High-intensity US 90 s.	7.7407 × 10^−5^	0.24091	0.90982
	High-intensity US 180 s.	0.044232	0.0083252	1.0327
	Medium-intensity US 90 s.	0.01738	0.015071	0.24005

**Table 3:** Hemolysis test measurement parameters.

Mode	Absorbance
Wavelength	414 nm
Bandwidth	9 nm
Reference Wavelength	600 nm
Number of Flashes	25
Settle Time	0 ms

**Table 4:** The hemolysis test results.

Blood Samples	Ultrasound Exposure Modes							
	1	2	3	4	5	6	7	8
A	3.4618	3.3851	OVER	OVER	OVER	0.3345	0.7347	
B	2.6052	OVER	OVER	OVER	OVER	0.9959	0.7763	
C	3.7265	OVER	OVER	OVER	OVER	0.4482	0.5144	
D	0.7124	0.7192	0.7573	0.7399	0.7399	0.2774	0.2485	0.0036
E	0.6673	0.6954	0.7192	0.7216	0.7216	0.2614	0.2475	0.004
F	0.5479	0.7046	0.7142	0.6945	0.6945	0.2518	0.2434	0.0047

## Data Availability

All commands and pipelines used in data processing were executed according to the manual and protocols of the corresponding medical software and MATLAB. No specific code has been developed for this study.
